# Quantifying behavioural interactions between humans and mosquitoes: Evaluating the protective efficacy of insecticidal nets against malaria transmission in rural Tanzania

**DOI:** 10.1186/1471-2334-6-161

**Published:** 2006-11-10

**Authors:** Gerry F Killeen, Japhet Kihonda, Edith Lyimo, Fred R Oketch, Maya E Kotas, Evan Mathenge, Joanna A Schellenberg, Christian Lengeler, Thomas A Smith, Chris J Drakeley

**Affiliations:** 1 Ifakara Health Research and Development Centre, Box 53, Ifakara, Morogoro, United Republic of Tanzania; 2 Department of Public Health and Epidemiology, Swiss Tropical Institute, Socinstrasse 57, Basel, CH 4002, Switzerland; 3 School of Biological and Biomedical Sciences, Durham University, Durham DH1 3LE, UK; 4 Faculty of Health Sciences, Moi University, P.O Box 4606, Eldoret, Kenya; 5 Department of Biomedical Engineering, Yale University, P.O. Box 208284; New Haven, CT 06520-8284, USA; 6 Department of Zoology, University of Nairobi, PO Box 30197, Nairobi, Kenya; 7 Department of Infectious and Tropical Diseases, London School of Hygiene and Tropical Medicine, Keppel Street, London, WC1E 7HT, UK

## Abstract

**Background:**

African malaria vectors bite predominantly indoors at night so sleeping under an Insecticide-Treated Net (ITN) can greatly reduce malaria risk. Behavioural adaptation by mosquitoes to increasing ITN coverage could allow vector mosquitoes to bite outside of peak sleeping hours and undermine efficacy of this key malaria prevention measure.

**Methods:**

High coverage with largely untreated nets has been achieved in the Kilombero Valley, southern Tanzania through social marketing programmes. Direct surveys of nightly biting activity by *An. gambiae *Giles were conducted in the area before (1997) and after (2004) implementation of ITN promotion. A novel analytical model was applied to estimate the effective protection provided by an ITN, based on published experimental hut trials combined with questionnaire surveys of human sleeping behaviour and recorded mosquito biting patterns.

**Results:**

*An. gambiae *was predominantly endophagic and nocturnal in both surveys: Approximately 90% and 80% of exposure occurred indoors and during peak sleeping hours, respectively. ITNs consistently conferred >70% protection against exposure to malaria transmission for users relative to non-users.

**Conclusion:**

As ITN coverage increases, behavioural adaptation by mosquitoes remains a future possibility. The approach described allows comparison of mosquito biting patterns and ITN efficacy at multiple study sites and times. Initial results indicate ITNs remain highly effective and should remain a top-priority intervention. Combined with recently developed transmission models, this approach allows rapid, informative and cost-effective preliminary comparison of diverse control strategies in terms of protection against exposure before more costly and intensive clinical trials.

## Background

The efficacy of insecticide-treated nets (ITNs) for preventing malaria is well established and they are known to provide substantial protection to both individuals and communities that use them [1,2]. The use of ITNs to prevent malaria in Africa is an excellent example of an intervention choice that is tailored to the context-specific ecology of the mosquito species responsible for transmission: The most important vectors of malaria in sub-Saharan Africa all bite predominantly indoors in the middle of the night so that sleeping under a treated net during this period can greatly reduce exposure to malaria transmission [3-6]. Recent reports from parts of northern Tanzania, where ITNs have been used for several years, suggests a subtle shift in mosquito behaviour to bite more frequently during hours of the early evening and early morning when people are more likely to be awake and vulnerable outside of their nets [7]. Although the selection of corresponding heritable behavioural traits has never been demonstrated to our knowledge, changes in mosquito biting habits can be immediately and directly induced by indoor residual spraying (IRS) of excito-repellent insecticides that prevent endophagy and delay feeding [6,8]. A range of national strategies based on targeted subsidies and public-private partnerships [9] are now being translated into growing levels of ITN coverage in many countries across malaria-endemic Africa [10]. In the Kilombero Valley, southern Tanzania, a long-running programme for social marketing of ITNs [11] has achieved 75% coverage in terms of use amongst the entire population (Killeen et al. Unpublished) and this approach has been incorporated into the ITN strategy of the National Malaria Control Programme [12]. While owning and using an ITN has clearly been shown to protect individuals in this context [13,14], these high levels of coverage have not dramatically reduced community-level transmission intensity experiences by non-users (Killeen et al, Unpublished). We therefore sought to determine whether changing biting patterns of local mosquito populations may have contributed to this lack of impact or whether the observed low levels of insecticide treatment [15] alone could explain these findings.

## Methods

### Study area

The epidemiology of malaria in the Kilombero Valley has been well described and a number of malaria control interventions, notably the KINET social marketing program for ITNs, have been evaluated in this setting [13,14,16-18]. The malaria transmission systems of this valley, and the village of Namwawala in particular, have been very well characterized, yielding a rich set of vector and parasite biodemographic parameters for detailed transmission modelling [19]. This low-lying, flooding river valley has historically experienced very high transmission intensities, typically ranging from 200 to 600 infectious bites per person per year in the rural villages where the highest entomological inoculation rate (EIR) we are aware of has been reported at an estimated 2,700 infectious bites per year [20]. While coverage with nets in this area was negligible in terms of usage by children in 1997 [13], it had reached 75% by 2004 although the vast majority of nets were untreated ([15] and Killeen et al, unpublished).

### Mosquito collection and processing

Human landing catch assessments of the nightly biting behaviour of mosquitoes were conducted in 1997 before significant levels of net coverage had been achieved [13] and again in 2004 when three-quarters of the population used nets (Killeen et al, Unpublished). All mosquitoes were first identified to sex and species based on morphological criteria and then classified visually as being unfed, part-fed, fed or gravid [3,21]. As is typical of sampling methods for host-seeking mosquitoes [22], the vast bulk of the catch were unfed (Killeen et al., unpublished) but all trapped mosquitoes were considered to be host-seeking.

In November 1997, four catchers conducted 12-hour human landing catches for 12 nights at two typical rural house in the village of Njagi in Kilombero District [23,24]. Each night one catcher caught mosquitoes immediately outside the house while the other conducted simultaneous catches inside the house. Every night, the catchers within each pair were exchanged between indoor and outdoor stations.

In July 2004, two catchers conducted 12-hour human landing catches for 24 nights distributed over one month at a typical rural house in the village of Lupiro in Ulanga district, Morogoro region [25]. Each night one catcher caught mosquitoes immediately outside the house while the other conducted simultaneous catches inside the house. Catching was only conducted for 45 minutes each hour, allowing 15 minutes rest with tea and snacks provided to maintain the alertness and motivation of the catchers. Total catches for each hour were divided by 0.75 to estimate the biting rate for a full hour.

### Human behavioural surveys

The proportion of time spent outdoors at each time point was estimated from answers to questionnaires, collected from 398 households during surveys of community-level transmission intensity between 2002 and 2004 (Killeen et al, Unpublished), in which people indicated the time they usually went to bed and arose in the morning.

### Estimating the personal protection provided to users by insecticide-treated nets

EIR is the product of the biting rate experienced by humans exposed to a vector population and the sporozoite infection prevalence of that mosquito population. The latter is only reduced by community-level impacts of malaria interventions [19,26] so here we estimate personal protection purely in terms of biting rates and the impact that measures such as ITNs have upon them. First we calculate B_u, t_, the mean biting rate experienced by an unprotected individual at each time of the night (t), based on the proportion of time spent outdoors multiplied by the outdoor biting rate at that time (B_o, t_) plus the proportion of that hour spent indoors multiplied by the indoor biting rate at that time (B_i, t_). The estimated proportion of people in bed sleeping or trying to sleep (S_t_) can be used to calculate the proportion of time spent indoors and outdoors each hour and thus the biting rate experienced:

B_u, t _= B_o, t _(1-S_t_) + B_i, t _S_t _    **1**

The number of bites experienced per night, or nightly biting rate, for an unprotected non-user (B_u_) can thus be calculated by summing the relevant biting rates for each hour:

Bu=∑t=124Bu,t     2
 MathType@MTEF@5@5@+=feaafiart1ev1aaatCvAUfKttLearuWrP9MDH5MBPbIqV92AaeXatLxBI9gBaebbnrfifHhDYfgasaacH8akY=wiFfYdH8Gipec8Eeeu0xXdbba9frFj0=OqFfea0dXdd9vqai=hGuQ8kuc9pgc9s8qqaq=dirpe0xb9q8qiLsFr0=vr0=vr0dc8meaabaqaciaacaGaaeqabaqabeGadaaakeaacqqGcbGqdaWgaaWcbaGaeeyDauhabeaakiabg2da9maaqahabaGaeeOqai0aaSbaaSqaaiabbwha1jabbYcaSiabbsha0bqabaaabaGaeeiDaqNaeyypa0JaeGymaedabaGaeGOmaiJaeGinaqdaniabggHiLdGccaWLjaGaaCzcaGqabiab=jdaYaaa@3F1F@

Note that an unprotected individual is defined as someone lacking any net whereas a protected individual is defined as someone regularly using an effectively insecticidal net. We model the nightly biting rate of a protected individual (B_p_) based on the combined nightly profiles of mosquito biting rate (B_u, t_) over time (t), the protective efficacy of ITNs (P), which is assumed to be constant, and the behaviour of humans which results in fluctuating adherence of ITN users over the course of the night. In the absence of more detailed behavioural surveys, the effective adherence of ITN use at a given time of the night was assumed to be equivalent to the proportion of people sleeping at that time (S_t_). This assumption allows us to express the overall effect of this interaction as follows:

Bp=∑t=124Bp,t=∑t=124[Bo,t(1−St)+Bi,tSt(1−P)]     3
 MathType@MTEF@5@5@+=feaafiart1ev1aaatCvAUfKttLearuWrP9MDH5MBPbIqV92AaeXatLxBI9gBaebbnrfifHhDYfgasaacH8akY=wiFfYdH8Gipec8Eeeu0xXdbba9frFj0=OqFfea0dXdd9vqai=hGuQ8kuc9pgc9s8qqaq=dirpe0xb9q8qiLsFr0=vr0=vr0dc8meaabaqaciaacaGaaeqabaqabeGadaaakeaacqqGcbGqdaWgaaWcbaGaeeiCaahabeaakiabg2da9maaqahabaGaeeOqai0aaSbaaSqaaiabbchaWjabbYcaSiabbsha0bqabaaabaGaeeiDaqNaeyypa0JaeGymaedabaGaeGOmaiJaeGinaqdaniabggHiLdGccqGH9aqpdaaeWbqaaiabcUfaBjabbkeacnaaBaaaleaacqqGVbWBcqqGSaalcqqG0baDaeqaaOGaeiikaGIaeGymaeJaeyOeI0Iaee4uam1aaSbaaSqaaiabbsha0bqabaGccqGGPaqkcqGHRaWkcqqGcbGqdaWgaaWcbaGaeeyAaKMaeeilaWIaeeiDaqhabeaakiabbofatnaaBaaaleaacqqG0baDaeqaaOGaeiikaGIaeGymaeJaeyOeI0IaeeiuaaLaeiykaKIaeiyxa0faleaacqqG0baDcqGH9aqpcqaIXaqmaeaacqaIYaGmcqaI0aana0GaeyyeIuoakiaaxMaacaWLjaacbeGae83mamdaaa@62D3@

Based on existing evidence from experimental hut trials [27-29], we assume a conservative minimum protective efficacy level of 80% for ITNs (P = 0.8), equivalent to a relative exposure to bites of 20% *when and only when actually sleeping under the net*: During waking hours, most residents were assumed to have remained outdoors and to have been exposed to the measured outdoor biting density whereas sleeping hours were presumed to be spent indoors and under an ITN if available.

Taking this available data for nightly human and mosquito behaviour profiles, we were therefore able to estimate the relative biting rate for ITN users which is equivalent to relative availability (λ_p_) as previously defined (See equations 8 and 14 in reference [19]). We calculate λ_p _by comparing the total biting rate that protected individuals are exposed to (B_p_) with that of non-users (B_u_) who are unprotected:

λ_p _= B_p_/B_u _    **4**

The *true *protective efficacy of an ITN(P*) against transmission exposure is then calculated as the overall nightly reduction of biting rate:

P* = 1 - λ_p _    **5**

This estimate of protective efficacy differs from that previously reported from experimental hut trials because it allows for typical shortcomings in adherence resulting from the time people typically spend outside of their ITN and indeed outside of the house. Note, however, that this estimate is merely a comparison between the biting rates experienced those who use an ITN and those who do not. Therefore, it does not include the community-level protection of both groups when ITNs reach sufficient levels of coverage to reduce vector biting densities and sporozoite prevalence over large areas [19].

A distinct and useful pair of indicators with which to interpret the results of the above equations are the proportion of exposure which occur indoors and the proportion that occurs during sleeping hours. The proportion of bites that occur during the observed peak sleeping hours of 9 pm to 5 am (π_s_) for an unprotected individual can thus be calculated as the nightly biting rate experienced during these hours divided by the total nightly biting rate:

πs=∑t=9 pm5 amBu,t/∑t=124Bu,t     6
 MathType@MTEF@5@5@+=feaafiart1ev1aaatCvAUfKttLearuWrP9MDH5MBPbIqV92AaeXatLxBI9gBaebbnrfifHhDYfgasaacH8akY=wiFfYdH8Gipec8Eeeu0xXdbba9frFj0=OqFfea0dXdd9vqai=hGuQ8kuc9pgc9s8qqaq=dirpe0xb9q8qiLsFr0=vr0=vr0dc8meaabaqaciaacaGaaeqabaqabeGadaaakeaacqaHapaCdaWgaaWcbaGaee4Camhabeaakiabg2da9maaqahabaGaeeOqai0aaSbaaSqaaiabbwha1jabbYcaSiabbsha0bqabaaabaGaeeiDaqNaeyypa0JaeGyoaKJaeeiiaaIaeeiCaaNaeeyBa0gabaGaeGynauJaeeiiaaIaeeyyaeMaeeyBa0ganiabggHiLdGccqGGVaWldaaeWbqaaiabbkeacnaaBaaaleaacqqG1bqDcqqGSaalcqqG0baDaeqaaaqaaiabbsha0jabg2da9iabigdaXaqaaiabikdaYiabisda0aqdcqGHris5aOGaaCzcaiaaxMaaieqacqWF2aGnaaa@535F@

Note that this value has previously been denoted C*, described as the proportion of human exposure during which an ITN is in use, and used as a key parameter for modelling the community- and individual-level effects of ITNs upon malaria transmission [19].

The proportion of bites occurring indoors (π_i_) for an unprotected individual can be calculated as the estimated number of bites estimated to occur indoors, divided by the total number of bites estimated to occur both indoors and outdoors:

πi=∑t=124[Bi,tSt]/∑t=124[Bo,t(1−St)+Bi,tSt]     7
 MathType@MTEF@5@5@+=feaafiart1ev1aaatCvAUfKttLearuWrP9MDH5MBPbIqV92AaeXatLxBI9gBaebbnrfifHhDYfgasaacH8akY=wiFfYdH8Gipec8Eeeu0xXdbba9frFj0=OqFfea0dXdd9vqai=hGuQ8kuc9pgc9s8qqaq=dirpe0xb9q8qiLsFr0=vr0=vr0dc8meaabaqaciaacaGaaeqabaqabeGadaaakeaacqaHapaCdaWgaaWcbaGaeeyAaKgabeaakiabg2da9maaqahabaGaei4waSLaeeOqai0aaSbaaSqaaiabbMgaPjabbYcaSiabbsha0bqabaGccqqGtbWudaWgaaWcbaGaeeiDaqhabeaakiabc2faDbWcbaGaeeiDaqNaeyypa0JaeGymaedabaGaeGOmaiJaeGinaqdaniabggHiLdGccqGGVaWldaaeWbqaaiabcUfaBjabbkeacnaaBaaaleaacqqGVbWBcqqGSaalcqqG0baDaeqaaOGaeiikaGIaeGymaeJaeyOeI0Iaee4uam1aaSbaaSqaaiabbsha0bqabaGccqGGPaqkcqGHRaWkcqqGcbGqdaWgaaWcbaGaeeyAaKMaeeilaWIaeeiDaqhabeaakiabbofatnaaBaaaleaacqqG0baDaeqaaOGaeiyxa0faleaacqqG0baDcqGH9aqpcqaIXaqmaeaacqaIYaGmcqaI0aana0GaeyyeIuoakiaaxMaacaWLjaacbeGae83naCdaaa@6402@

These analyses and the model used to execute them are available in Microsoft Excel spreadsheet format as an online supplement number to this paper [see Additional file 1].

### Extrapolation of individual protection to community level suppression of transmission

The estimates of effective adherence (π_s_) and true personal protective P* described above are key parameters in a recently developed model of malaria transmission intensity as a function of coverage with personal protection measures [19]. As previously described (except that it was previously denoted C*), π_s _was set to a value of 0.80, reflecting the results reported here. Parameters reflecting the diversionary and killing properties of ITNs, respectively denoted Δ_p _and μ_p _[19], were both tuned to 0.60 to yield a predicted value for P* that matches the mean field estimate of 0.73 reported here (See results). This level of personal protection was then extrapolated to predict community-level impacts of varying coverage levels in vector systems dominated by *An. gambiae *assuming a daily survival rate of 0.80 for foraging mosquitoes [19]. For comparison, alternative larviciding strategies were modelled by simply assuming that the emergence rate and hence biting density of vectors is equivalent to coverage of the aquatic-stage mosquito population [30,31]. These simulations and the model used to execute them are available in Microsoft Excel spreadsheet format [see Additional file 1].

### Protection of human subjects and ethical approval

All participants as mosquito catchers in this study were provided with access to the best treatment available at the time (sulphadoxine-pyrimethamine in 1997, artemether-lumefantrine in 2004) and weekly screening for malaria parasites by light microscopy. Both studies were approved by the Medical Research Coordination Committee of the National Institute for Medical Research of the United Republic of Tanzania (Reference numbers NIMR/HQ/R8a/Vol VIII/1, NIMR/HQ/R.8a/VOL.IX/324 and NIMR/HQ/R.8a/VOL.X/12)

## Results

**Figure 1:**
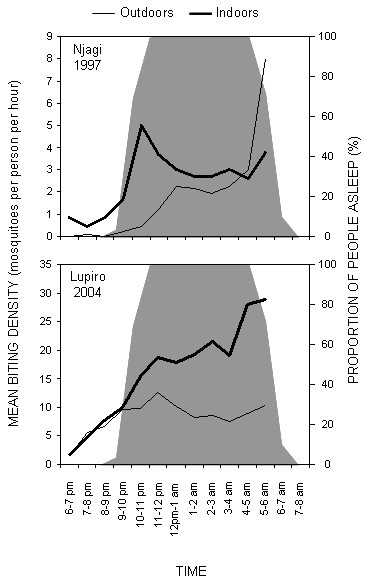
Mean indoor and outdoor biting densities of *An. gambiae *at two sites in the Kilombero Valley in 1997 and 2004. Grey background shading represents the proportion of the human population in bed. Note these estimates reflect the biting rate experienced by a human landing catcher sampling exclusively indoors or outdoors, rather than the calculated biting rates experienced by a typical resident that are presented in figures 2 to 4 [see Additional file 1].

*An. gambiae s.l*. was the only major malaria vector caught in sufficient numbers at both sites to allow comparison of biting behaviour before and after the implementation of the KINET project. The sibling species composition of this complex at both sites is strongly predominated by *An. gambiae sensu stricto *[23,25]. Although Lupiro in 2004 had a somewhat different peak of biting, compared with Njagi in 1997 (Figure 1), both surveys report biting patterns which are consistent with the range of biting patterns historically reported for *An. gambiae *[3-6]. In 1997 a peak of indoor biting occurred in the early evening at Njage and again at dawn as previously described [3]. By comparison, Lupiro in 2004 witnessed a steady increase of indoor activity right up until dawn, remarkably similar to the laboratory observations of biting activity [32] quoted by Gilles and DeMellion's classic monograph [3].

**Figure 2:**
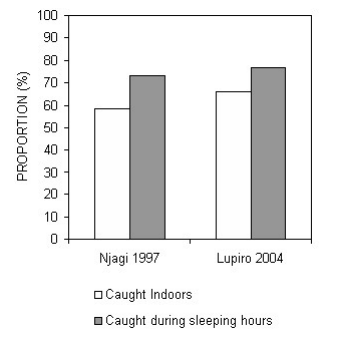
Proportion of all *An. gambiae *caught indoors and during peak sleeping hours (9 pm to 5 am) at two sites in the Kilombero Valley in 1997 and 2004. Standard errors are not plotted as they are consistently < 0.001 for all estimates, n = 674 and 5,931 in 1997 and 2004, respectively [see Additional file 1].

Although the 2004 surveys yielded significantly higher proportions of *An. gambiae *mosquitoes caught indoors and during sleeping hours (P = 0.0001 and P = 0.023 by Χ^2 ^test, respectively), the magnitude of these subtle differences are normal for this species in different times and places [3]. Overall, both surveys describe predominantly endophagic (See references [6] and [8] for lucid definitions) and nocturnal populations of *An. gambiae*. Even the statistically significant but epidemiologically negligible differences between the two surveys are opposite to that would be expected if behavioural adaptation had in fact occurred (Figure 2). Given that this particular vector population is known to exhibit consistent biting habits independently of population density [33], we conclude that these small differences between the two surveys can be readily explained in terms of normal household-scale behavioural plasticity in response to the meteorological conditions and the microenvironments presented by the limited number of houses which were sampled.

**Figure 3:**
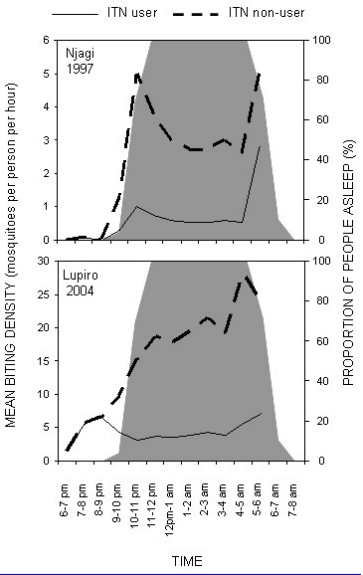
Estimated patterns of exposure to biting *An. gambiae *for ITN users and non-users at two sites in the Kilombero Valley in 1997 and 2004. Grey background shading represents the proportion of the human population in bed [see Additional file 1].

**Figure 4:**
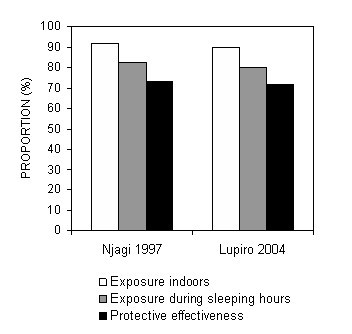
Estimated proportions of exposure to biting *An. gambiae *occurring indoors (π_i _; equation **7**) and during peak sleeping hours (π_s _; equation **6**)for non-users of ITNs as well as the estimated true protective efficacy of ITNs (P*; equation **5**), for ITN users at two sites in the Kilombero Valley in 1997 and 2004 [see Additional file 1].

Based on the results of the human behaviour surveys, we estimate a mean of 8.5 hours spent in bed each night. Most people typically slept indoors between 9 pm and 5 am when the bulk of mosquito biting activity occurs (Figure 3). Even though the early peak of biting activity observed in 1997 comes just before most people go to bed, the bulk of exposure estimated in both surveys occurs indoors and during sleeping hours (Figure 4). Approximately 90% and 80% exposure occurred indoors and during peak sleeping hours, respectively in both surveys. In 1997 and 2004, an ITN is respectively estimated to provide 71.7 and 73.3% true protective efficacy to routine users against exposure to *An. gambiae *biting activity and inoculation with malaria parasites (Figure 4). Assuming the Abuja targets of at least 60% coverage with effectively insecticidal nets are eventually realized and this were to apply to the entire population, *An. gambiae *mosquitoes would encounter a potentially fatal ITN on 48% of all attempts to feed upon humans (80% of bites normally occurring on users would occur at times when an ITN is in use × 60 % usage of ITNs).

**Figure 5:**
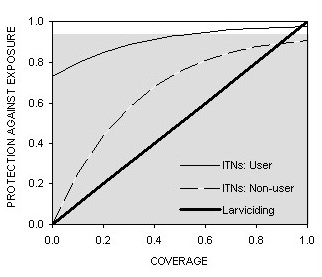
Predicted protection against exposure resulting from increasing coverage of either human populations with ITNs or aquatic stage vector populations with larvicides. Grey shading represents the level of protection recently reported for systematic application of microbial larvicides in rural Kenya [34]. The model [19] and input parameters are available for examination in the excel spreadsheet used to generate this figure [see Additional file 1].

In order to demonstrate the utility of the integrated field surveys and analytical approach we present here, we provide an example comparing the existing gold standard of ITNs with emerging larviciding strategies: As described in the methods section, combining this approach with emerging process-explicit transmission models [19] allows simulated comparison of ITNs with quite different interventions that have a more direct impact upon vector emergence rates and densities at source. Taking the 92% reduction of exposure recently achieved with biological larvicides in rural Kenya [34] as a benchmark, equivalent protection for ITN users is predicted at a coverage of 48% use in the entire population (Figure 5). ITNs achieve good levels of protection for non-users at modest coverage levels and approach the equitable protection afforded by larviciding at very high population coverage. Interestingly, this communal and equitable protection has a high but finite limit because of diversion to the few remaining unprotected hosts at high coverage [19]. This analysis supports the view that both larviciding and ITNs can achieve worthwhile and equitable impacts for all members of human populations if high coverage can be realized [31,34-43] while the latter can also deliver useful individual protection, regardless of coverage [1,19].

## Discussion

Based on the nocturnal biting patterns reported here, we find no evidence for adaptation by *An.gambiae *to high coverage of nets across the Kilombero Valley. The small proportion of nets in Kilombero which are actually treated with insecticide still provide effective individual protection against exposure to malaria, consistent with the results of cohort studies of child mortality in the area [13] and similar entomological studies in parts of west Africa [44]. Unfortunately, the protective efficacy we estimate for ITNs in this setting is only likely to be achieved by the minority of users in the Kilombero Valley whose nets are actually insecticidal: Less than 10% of nets in this area were reported to have been treated in the last year and confirmed to contain sufficient levels of insecticide [15] while the remainder are expected to provide little if any protection [6,27-29].

At the low levels of coverage with truly insecticidal nets observed in Kilombero [15], this analysis predicts little community-level protection of non-users (Figure 5), consistent with the results of large scale field surveys during this period (Killeen et al, Unpublished). It is therefore imperative that long-lasting nets and net treatments [27,28,45-48] are rapidly incorporated into local, national and international initiatives [9,12] to increase coverage with nets which are treated, kill mosquitoes and prevent malaria more effectively. If levels of coverage with ITNs can be achieved that compare with existing coverage with largely untreated nets, we anticipate this will result in massive reductions of malaria transmission and burden, for users and non-users alike [19,43,49]. If the Abuja targets are eventually realized for entire populations rather than just vulnerable groups, this will place unprecedented levels of direct behavioural stimulation and longer-term selective pressure upon the major vectors of malaria of Kilombero and elsewhere in Africa. We therefore caution that although little evidence is yet available for behavioural adaptation to the presence of ITNs, it has been recently reported [7] and remains a possibility for the future.

This study has several limitations which can be improved upon. These estimates for the individual protective efficacy of an ITN may be slightly exaggerated because mosquitoes were not sampled between 6 am and 7 am when a surge in biting activity can occur [3]. This seems particularly likely given the observed increase in biting activity that was observed as dawn approached in both surveys (Figure 1). Future studies should therefore include this short but important period. Although this analysis was conducted retrospectively with existing data, future studies could include repeat the surveys of human behaviour at each time interval. Such studies could also be designed to consider time spend indoors but not under an ITN, the presence of additional relevant interventions and variations in these factors according to intervention availability, socioeconomic status, meteorology and season [50].

Accepting these shortcoming, this novel estimate compares well with recent studies in northern Tanzania that combine established domestic trapping methodologies with genetic fingerprinting of human blood meals to estimate individual protection against exposure of 69% [51]. It is also approximately consistent with previous calculations of protection based on the assumption that ITNs are entirely mosquito proof [6]. One advantage of the approach described here is that it allows rationalization in terms of directly observable behaviours of mosquitoes and humans, as well as comparison of these patterns across diverse settings and timescales. Furthermore, it allows estimation of the proportion exposure which occurs outdoors and therefore cannot be prevented through direct individual protective effects of domestic interventions such as ITNs [1], indoor residual application of insecticides [49,52,53] or house screening [54,55]. Conventional experimental hut trials [27-29] usually consider protection afforded when ITNs are actually in use and, even when applied under typical village conditions [51], can only consider protection against indoor exposure. In contrast, this analysis is the first attempt we are aware of to estimate the true individual protective efficacy of ITNs against mosquitoes by allowing for exposure occurring when ITN users are not asleep and not protected. It can therefore also be readily applied to settings where vectors are primarily exophagic and even to interventions that act outdoors, such as topical repellents [56] or insecticide-treated clothing [57,58]. While this approach is quite inexpensive, with the 2004 field survey described here costing approximately US$7,000, the largest disadvantage is undoubtedly the necessity conduct human landing catches and exposes participants to increased malaria transmission hazard [22]. Thus the considerable advantages and disadvantages of this approach, as well as the balance of risks and benefits should be carefully reviewed and justified before application.

Here we estimate that 10% of exposure of a person lacking an ITN occurs outdoors, setting a ceiling for the direct individual protective effects of such measures. It therefore follows that over a third of transmission experienced by an ITN user cannot be prevented by the individual effects of further domestic interventions. Thus to achieve further reductions of disease burden, integrated vector management programmes should aim to maximize community-level effects of such domestic measures by increasing population level coverage because these will protect all members of the community at all times, regardless of whether they are personally covered [19]. In order to achieve such equitable community-wide impacts and improve levels of individual protection, coverage with domestic personal protection may be complemented with other measures such as repellents which protect individuals while outdoors or outside of their ITN [56,57,59,60]. Additional options for reducing communal malaria transmission include large-area vector control measures which reduce the transmission potential of entire local vector populations through either larval control [34,35,61] or insecticide treatment of alternative hosts [62,63]. These may be augmented further with clinical interventions which suppress human infectiousness with gametocidal drugs or transmission-blocking vaccines [64].

The estimate for ITN personal protection we present here compares very well with estimates of 72% for household-level protection against *An. gambiae *inside houses treated with dichlorodiphenyltrichloroethane (DDT) [8,65], consistent with the view that these are equally efficacious front-line interventions [49]. It therefore follows that to achieve comparable impacts, other transmission control interventions, including larval control [35,61], insecticide treatment of alternative hosts [62,63] and transmission-blocking vaccines or drugs [64] should aim to reduce human exposure to a similar degree and ideally do so at a comparable cost per person protected per unit time.

In addition to such direct applications as measuring individual protective effects of measures like ITNs, this approach also allows their impact upon community-level exposure to be predicted. By combining the analysis described above with recently developed models of malaria transmission and epidemiology [19,66], it is possible to compare a variety of other vector control measures with this widely accepted gold standard. Although the majority of ITN evaluations estimate their individual protective effects only, they are consistently efficacious in a variety of settings across Africa [1] and achieve cost-effectiveness equivalent to childhood vaccination [12]. Substantial impacts upon disease burden are experienced by individual ITN users, even in this holoendemic setting [13,14], and these results indicate such impacts are achieved through a 72.5% reduction of exposure to transmission. Thus even this partial personal protection against the massive exposure levels that characterize much of sub-Saharan Africa [67,68] delivers worthwhile alleviation of malaria infection and disease burden [1]. This impact appears somewhat greater than might be expected based on the relationship between transmission intensity and malaria burden in differing geographic areas [67,68]. We suggest that this discrepancy is most likely explained by the difference between immediately impacts of transmission suppression and those seen in the long term following re-equilibration of transmission intensity, force of infection and population immunity [69]. Nevertheless, we conclude that any intervention which affordably suppresses exposure to transmission by proportions similar to that delivered by an ITN to a single user in a population merits consideration for detailed field trials and epidemiological simulations to determine their likely cost effectiveness in the long term [69,70].

## Conclusion

As ITN coverage increases all across Africa, regular assessments of mosquito biting patterns should be conducted, ideally in sites where historical data allows the identification of trends over time. The approach described here for quantifying behavioural interaction between mosquitoes and humans can be applied to such monitoring activities and enable comparisons of multiple study sites over time. Such direct and plausible estimates of protection against malaria exposure could allow more precise study of the relationship between transmission exposure and consequent risk of infection, disease or death [66,67,71] where personal protection measures are common. Although the efficacy and effectiveness of various control strategies must clearly be field-evaluated in terms of their impact upon morbidity and mortality, the approach described here allows rapid preliminary comparison of their diverse transmission suppression effects at minimal cost. This is because this methodology allows relatively direct comparison of diverse transmission control strategies in terms of their impact on human exposure, rather than force of infection or incidence of disease. We propose that such entomological evaluations should treat ITNs and IRS as gold standards against which the protective affects of alternatives can be compared. While estimates of protection against exposure are no substitute for careful prospective morbidity and mortality studies, they do allow informed evaluation of transmission control methods and cost-effective selection of those most likely to justify investment in large scale demographic and clinical surveillance systems. Application to this setting indicates that ITNs remain highly efficacious and should remain a top-priority option for malaria control in even the most isolated and resource-limited settings. Ongoing efforts to scale up the coverage and quality of this essential intervention [9] should proceed with maximum support from all sectors.

## Authors' contributions

GFK conceived the 2004 entomological survey, formulated the models and wrote the manuscript in consultation with the other authors. JK, EL, FRO and MEK conducted the entomological and human behavioural surveys and contributed to the drafting of the manuscript. EM advised upon the design of the 2004 entomological survey. JAS, CL, TAS and CJD designed and supervised the various studies these additional mosquito surveys were embedded within and contributed substantially to the drafting of the manuscript. TAS and CJD also contributed to the refinement of the models.

## Competing interests

The author(s) have no competing interests relating to this research

## Pre-publication history

The pre-publication history for this paper can be accessed here:



## Supplementary Material

Additional File 1 1; Human-mosquito interaction 1997, 2; Human-mosquito interaction 1997 and 3; Strategy comparison. This Excel spreadsheet presents (1 and 2) the actual data and analytical model used to estimate the personal protection afforded by an ITN and (3) an integrated transmission model extrapolating these individual effects to entire populations and comparing these impacts with larviciding. Click here for file
